# Difference in dose‐volumetric data between the analytical anisotropic algorithm, the dose‐to‐medium, and the dose‐to‐water reporting modes of the Acuros XB for lung stereotactic body radiation therapy

**DOI:** 10.1120/jacmp.v17i5.6338

**Published:** 2016-09-08

**Authors:** Wambaka A. Mampuya, Mitsuhiro Nakamura, Yoshinori Hirose, Kenji Kitsuda, Takashi Ishigaki, Takashi Mizowaki, Masahiro Hiraoka

**Affiliations:** ^1^ Department of Radiation Oncology and Image‐applied Therapy Graduate School of Medicine, Kyoto University Sakyo‐ku Kyoto Japan; ^2^ Division of Radiology Osaka Red Cross Hospital Osaka Japan; ^3^ Department of Radiation Oncology Osaka Red Cross Hospital Osaka Japan

**Keywords:** Acuros XB, dose‐to‐water ($D_{w}$), dose‐to‐medium ($D_{m}$), AAA (anisotropic analytical algorithm), dose‐volumetric data, stereotactic body radiation therapy

## Abstract

The purpose of this study was to evaluate the difference in dose‐volumetric data between the analytical anisotropic algorithms (AAA) and the two dose reporting modes of the Acuros XB, namely, the dose to water (${\text{AXB}}_{-}D_{w}$) and dose to medium (${\text{AXB}}_{-}D_{m}$) in lung stereotactic body radiotherapy (SBRT). Thirty‐eight plans were generated using the ${\text{AXB}}_{-}D_{m}$ in Eclipse Treatment Planning System (TPS) and then recalculated with the ${\text{AXB}}_{-}D_{w}$ and AAA, using identical beam setup. A dose of 50 Gy in 4 fractions was prescribed to the isocenter and the planning target volume (PTV) D95%. The isocenter was always inside the PTV. The following dose‐volumetric parameters were evaluated; D2%, D50%, D95%, and D98% for the internal target volume (ITV) and the PTV. Two‐tailed paired Student's t‐tests determined the statistical significance. Although for most of the parameters evaluated, the mean differences observed between the AAA, ${\text{AXB}}_{-}D_{m}$and ${\text{AXB}}_{-}D_{w}$ were statistically significant (p<0.05), absolute differences were rather small, in general less than 5% points. The maximum mean difference was observed in the ITV D50% between the ${\text{AXB}}_{-}D_{m}$ and the AAA and was 1.7% points under the isocenter prescription and 3.3% points under the D95 prescription. ${\text{AXB}}_{-}D_{m}$ produced higher values than ${\text{AXB}}_{-}D_{w}$ with differences ranging from 0.4 to 1.1% points under isocenter prescription and 0.0 to 0.7% points under the PTV D95% prescription. The differences observed under the PTV D95% prescription were larger compared to those observed for the isocenter prescription between ${\text{AXB}}_{-}D_{m}$ and AAA, ${\text{AXB}}_{-}D_{m}$ and ${\text{AXB}}_{-}D_{w}$, and ${\text{AXB}}_{-}D_{w}$ and AAA. Although statistically significant, the mean differences between the three algorithms are within 3.3% points.

PACS number(s): 87.55.x, 87.55.D‐, 87.55.dk

## I. INTRODUCTION

The field of radiation oncology has rapidly evolved over the last three decades due to the advances in diagnostic radiology that allows for better tumor volume definition during treatment planning, and the development of new irradiation techniques such as intensity‐modulated radiotherapy (IMRT) and stereotactic body radiation therapy (SBRT), making possible a better optimization of dose distribution around the planning target volume (PTV).^(1)^ Those advanced techniques require a high and verified accuracy in dose calculation to ensure that the prescribed dose is actually delivered to the tumor with good sparing of the surrounding normal tissues. This would result in an increased cure rates, as well as improved patients' tolerance to treatment.^(2)^ Indeed, according to the American Association of Physicists in Medicine (AAPM) Report No. 85, a 5% change in dose may induce a 10% to 20% change in tumor control probability (TCP) and a 20% to 30% change in complication rates of normal tissues (NTCP).^(3)^ Due to the high dose and small number of fractions used in SBRT, accuracy issue becomes of even greater importance.^(4)^

Monte Carlo (MC) dose calculation algorithm, which stochastically solves the Linear Boltzmann Transportation Equations (LBTE), is considered as the gold standard in terms of accuracy, especially in heterogeneous media such as lung.^(5)^ MC explicitly models the physical interaction of each particle in media, a process which remains time‐consuming despite recent advances in computing power. This explains why MC is still not widely used in daily clinical practice.^5^, ^6^ Recently, another dose calculation algorithm using a deterministic grid‐based Boltzmann equation solver has been licensed by Varian Medical Systems (Palo Alto, CA) and has been implemented in the Eclipse Treatment Planning System as the Acuros XB (AXB), advanced dose calculation algorithm.^(7)^ AXB has shown to agree very well with MC, even in heterogeneous media, with the advantage of having a faster calculation times and being available on a commercial TPS.^8^, ^9^

Like MC, AXB can report the absorbed dose in two modes: dose‐to‐water ($D_{w}$) and dose‐to‐medium ($D_{m}$). The $D_{w}$ has been widely used in conventional radiotherapy and several dosimetry protocols are based on $D_{w}$. However, recent trends are in the use of LBTE solver type algorithms, which can report dose to medium in addition to $D_{w}$. It should be noted however that, although the question of which dose reporting mode to use remains controversial,^(10)^ $D_{m}$ can be rescaled to $D_{w}$ using the stopping‐power ratio of water‐to‐medium. Siebers et al.^(11)^ evaluated water‐to‐medium mass collision stopping‐power ratios as a function of electron energy. They reported a substantial decrease in the water‐to‐medium stopping power for the lung with an increase in the monoenergetic electron energy, suggesting that the difference between $D_{m}$ and $D_{w}$ may increase in favor of the $D_{w}$ when it comes to the lung.

Recently several studies have compared the dose‐volumetric data obtained with ${\text{AXB}}_{-}D_{m}$, with either MC or the analytical anisotropic algorithm (AAA), but only a few studies have evaluated the difference in dose‐volumetric data between the two dose reporting modes of AXB in lung cancer patients treated with SBRT8.^12^, ^13^, ^14^, ^15^, ^16^, ^17^, ^18^ Rana and Pokharel^(16)^ reported that the selection of either $D_{m}$ or $D_{w}$ in AXB is less likely to produce significant dosimetric differences in the clinical environment. However, only five patients in their series were treated with SBRT for lung cancer, making it difficult to draw any conclusion from their study. The aim of the present study is to evaluate the difference in dose‐volumetric parameters between the ${\text{AXB}}_{-}D_{m}$ and ${\text{AXB}}_{-}D_{w}$ on a larger series of clinical patients treated with SBRT for lung cancer. Additionally, we also compared the dose‐volumetric data of either dose reporting mode of the AXB with the AAA.

## II. MATERIALS AND METHODS

### A. Treatment procedure

All patients underwent a respiration‐correlated 4D CT scan using the Varian Real Time Position Management Respiratory Gating System, version 1.7 (Varian Medical Systems) and a Discovery CT750HD CT Scanner (General Electric Medical Systems, Waukesha, WI) with a slice thickness of 2.5 mm in the axial cine mode.

Then, the 4D CT slices and respiratory motion data were transferred to an Advantage 4D Workstation (General Electric Medical Systems, San Francisco, CA), where maximum intensity projection (MIP) and averaged intensity projection (AIP) images were obtained after a phase binning of the 4D CT in 10 equally spaced phase bins. The dataset was imported to the Eclipse (Varian Medical Systems) for treatment planning using 6 MV photon beams. ITV was delineated on the AIP image with references to the MIP image. PTVs were created by adding 5‐mm margins to the ITVs in all directions.^(19)^ A dose of 50 Gy in 4 fractions was prescribed to the isocenter and the PTV D95%. The isocenter was always inside the PTV. Dose calculation was done with the default dose‐to‐medium reporting mode of the AXB (version 11.0.31) with a grid size of $2.5\times 2.5\times 2.5\;{\text{mm}}^{3}$. Recalculation was subsequently done with the ${\text{AXB}}_{-}D_{w}$ and AAA (version 11.0.31) using identical beam setup.

### B. Evaluated parameters

The dose received by n% volume of the target volume (ITV and PTV), D2%, D50%, D95%, and D98%, were evaluated. We compared the relative differences in the corresponding evaluated parameters between ${\text{AXB}}_{-}D_{m}$ and AAA, ${\text{AXB}}_{-}D_{m}$ and ${\text{AXB}}_{-}D_{w}$and ${\text{AXB}}_{-}D_{m}$ and AAA. The two‐sided, paired Student's *t*‐test was used to determine the statistical significance. Values of p<0.05 were regarded as significant.

## III. RESULTS

Thirty‐seven consecutive patients diagnosed with lung cancer and treated with SBRT from July 2011 to August 2015 were included in the present dosimetric study. One patient had two lesions, one in the right and one in left lung. In total, 38 treatment plans were developed. Table 1 summarized the dose‐volumetric data results under the isocenter and the PTV D95% prescription for all the 37 patients.

**Table 1:** Dose‐volumetric data calculated with ${\text{AXB}}_{-}D_{m}$, ${\text{AXB}}_{-}D_{w}$and AAA. Data are shown as mean ± standard deviation.

	*Isocenter (%)*			*PTV D95% (%)*		
	${\text{AXB}}_{-}D_{m}$	${\text{AXB}}_{-}D_{w}$	${\text{AXB}}_{-}D_{m}$	${\text{AXB}}_{-}D_{m}$	${\text{AXB}}_{-}D_{w}$	*AAA*
*PTV*						
D2%^e^	101.7±2.2	101.0±2.1	100.6±1.7	133.4±7.7	133.1±7.6	131.±6.5
D50%^a^	95.4±4.0	94.8±3.5	93.9±3.2	120.4±4.3	120.3±4.3	118.±4.3
D95%^d^	86.9±6.2	86.5±6.0	86.2±5.5	100.1±0.0	100.1±0.0	100.1±0.0
D98%^c^	84.6±7.0	84.2±6.8	84.2±6.5	93.8±2.4	93.8±2.4	94.2±2.6
*ITV*						
D2%^b^	102.1±1.9	101.1±1.6	101.±1.3	134.3±8.2	133.9±8.2	132.1±7.1
D50%^a^	98.6±2.2	97.7±1.8	96.9±1.9	128.3±7.4	127.6±7.3	125.0±6.4
D95%^a^	94.0±3.4	93.3±3.3	92.4±3.2	118.5±12.5	118.2±12.5	115.9±12.1
D98%^a^	92.7±5.0	92.±5.0	91.2±4.8	116.0±15.0	115.8±14.9	113.6±14.8

^a^ A significant difference was found between ${\text{AXB}}_{-}D_{m}$ and AAA, ${\text{AXB}}_{-}D_{m}$ and ${\text{AXB}}_{-}D_{w}$ and AAA and ${\text{AXB}}_{-}D_{w}$.

^b^ A significant difference was found between ${\text{AXB}}_{-}D_{m}$ and AAA, ${\text{AXB}}_{-}D_{m}$ and ${\text{AXB}}_{-}D_{w}$ and AAA and ${\text{AXB}}_{-}D_{w}$ only under the PTV D95% prescription.

^c^ A significant difference was found between ${\text{AXB}}_{-}D_{m}$ and AAA and AAA and ${\text{AXB}}_{-}D_{w}$ under the PTV D95% prescription.

^d^ A significant difference was found between ${\text{AXB}}_{-}D_{m}$ and AAA, ${\text{AXB}}_{-}D_{m}$ and ${\text{AXB}}_{-}D_{w}$ under the isocenter prescription.

^e^ A significant difference was found between ${\text{AXB}}_{-}D_{m}$ and AAA, ${\text{AXB}}_{-}D_{m}$ and ${\text{AXB}}_{-}D_{w}$ and AAA and ${\text{AXB}}_{-}D_{w}$ under the isocenter prescription and ${\text{AXB}}_{-}D_{w}$ and AAA and ${\text{AXB}}_{-}D_{w}$ under the PTV D95% prescription.

${\text{AXB}}_{-}D_{m}$ = Acuros XB dose‐to‐medium reporting mode; ${\text{AXB}}_{-}D_{w}\;=\text{Acuros XB}$ dose‐to‐water reporting mode; AAA= analytical anisotropic algorithm; PTV=planning target volume; ITV=internal target volume; D95%= prescription covering 95% of the target volume.

### A. Under the isocenter prescription

The maximum mean difference observed in the ITV D50% between the ${\text{AXB}}_{-}D_{m}$ and the AAA was only 1.7% points, although statistically significant (p<0.05). The difference in the PTV D98% was not statistically significant between the three algorithms with p=0.88,0.05, and 0.11 between ${\text{AXB}}_{-}D_{m}$ and AAA, ${\text{AXB}}_{-}D_{m}$ and ${\text{AXB}}_{-}D_{w}$and ${\text{AXB}}_{-}D_{m}$ and AAA, respectively.

### B. Under the PTV D95% prescription

**Figure 1:**
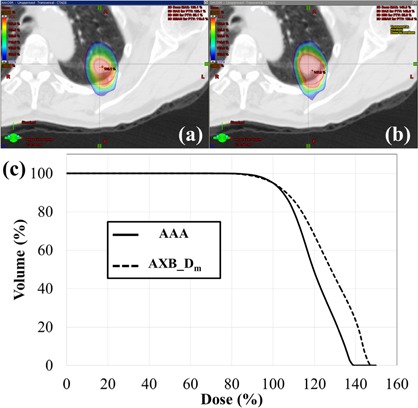
Representative dose distributions calculated with (a) AAA and (b) ${\text{AXB}}_{-}D_{m}$ in the axial plane, and (c) the corresponding dose‐volume histograms for the PTV. A dose of 50 Gy in 4 fractions was prescribed to the PTV D95%.

The maximum mean difference, observed in the ITV D50% between the ${\text{AXB}}_{-}D_{m}$ and the AAA was only 3.3% points, although statistically significant (p<0.05). The difference in the PTV D98% and D2% was not statistically significant between the ${\text{AXB}}_{-}D_{m}$ and ${\text{AXB}}_{-}D_{w}$ (*p* = 0.19 and 0.18, respectively). The PTV D95% didn't differ between the three algorithms. Figure 1 shows dose distributions and DVH for the patient with the largest difference between the ${\text{AXB}}_{-}D_{m}$ and the AAA. The percentage of the PTV receiving more than 130% of the prescribed dose with ${\text{AXB}}_{-}D_{m}$ was almost double of that with AAA. In this patient, difference in D2% and D50% were more than 8.6% and 8.5%, respectively.

## IV. DISCUSSION

LBTE solver type algorithm such a MC and AXB allows the expression of radiation transport and energy deposition in patient representative media. The dose reporting mode can then either be $D_{m}$ or $D_{w}$, the difference between the two in Acuros XB being the way dose is calculated. Indeed, when $D_{m}$ is calculated, the energy‐dependent response function is based on the material properties of that voxel. When $D_{w}$ is calculated, the energy‐dependent fluence‐to‐dose response function is based on water.^(20)^

The possibility, at first provided by Monte Carlo simulation, of reporting the dose as $D_{m}$, has become a subject of controversy in medical physics community. The pros and cons of using either of the dose reporting modes have been discussed in a point/counterpoint debates published in Medical Physics.^(10)^ In favor of using the $D_{m}$ is the arguments that a) converting $D_{m}$ back to $D_{w}$ requires a stopping power ratio, adding uncertainties and increasing MC calculation time; b) the clinical impact of switching from $D_{w}$ to $D_{m}$ is not expected to be significant; and c) use of $D_{m}$ allows the establishment of more accurate dose delivery, and provides a closer relationship between tissue response and dose. On the other hand, arguments favoring the use of $D_{w}$ included the following: a) clinical experience is $D_{w}$‐based; b) dosimetry protocols are $D_{w}$‐based; c) the “medium” to report dose in is always a guess since accompanying 3D body composition analysis is often absent; and d) clinical prescription can be achieved with $D_{w}$‐based IMRT.

In the present study, we evaluated the difference in dose‐volumetric data between the ${\text{AXB}}_{-}D_{w}$, ${\text{AXB}}_{-}D_{m}$and the AAA in a larger series of clinical patients treated for lung cancer with SBRT. Although for most of the parameters evaluated the mean differences observed between the AAA, ${\text{AXB}}_{-}D_{m}$and ${\text{AXB}}_{-}D_{w}$ were statistically significant, absolute differences were rather small, in general less than 5% points. Under both isocenter and PTV D95% prescription, the largest difference observed was between ${\text{AXB}}_{-}D_{m}$ and AAA in the ITV D50%, followed by the ITV D95% and ITV D98%; that was, in the ITV margin. This can be explained by the difference in radiation transport modeling between the AAA and the AXB. Furthermore, while the AAA computes the transport and dose deposition using radiological and density scaling, reports the absorbed dose as if it were deposited in water, but for both options of AXB, calculated dose considers the elemental composition of the specific medium. Doses calculated using ${\text{AXB}}_{-}D_{w}$ were generated by converting the doses calculated by ${\text{AXB}}_{-}D_{m}$ with the stopping‐power ratio for water to the specific medium. The mean difference for both the ITV D50% and the remaining parameters evaluated was higher between ${\text{AXB}}_{-}D_{m}$ and AAA than ${\text{AXB}}_{-}D_{w}$ and AAA. Siebers et al.^(11)^ reported that the stopping power tends to become small with an increase in the energy so that at 6 MV, the energy used in the present study, one could have expected that $D_{w}$ produce higher values than $D_{m}$. However, in our study, ${\text{AXB}}_{-}D_{m}$ produced higher values, and the mean differences between ${\text{AXB}}_{-}D_{w}$ and ${\text{AXB}}_{-}D_{m}$ showed a clear trend in favor of ${\text{AXB}}_{-}D_{m}$. Hence the larger differences observed between ${\text{AXB}}_{-}D_{m}$ and AAA than ${\text{AXB}}_{-}D_{w}$ and AAA.

Studies evaluating the difference in dose‐volumetric data between the two dose reporting modes of the AXB in lung SBRT patients are scarce. Rana and Pokharel^(16)^ evaluating five patients, found the difference between ${\text{AXB}}_{-}D_{m}$ and ${\text{AXB}}_{-}D_{w}$ to be patient‐specific without a clear trend, ranging from −1.4% to 2.9%. They concluded that the selection of either ${\text{AXB}}_{-}D_{m}$ or ${\text{AXB}}_{-}D_{w}$ is less likely to produce significant dosimetric differences in the clinical environment. In our study, using a larger series of clinical patients, we could observe a clear trend in favor of ${\text{AXB}}_{-}D_{m}$ with differences ranging from 0.4 to 1.1% points under isocenter prescription and 0.0 to 0.7% points under the PTV D95% prescription. This rather small difference could be explained by the fact that the electron transport is the same between ${\text{AXB}}_{-}D_{m}$ and ${\text{AXB}}_{-}D_{w}$and only the electron energy deposition interaction is different. Moreover, the AAPM Report No. 85^(21)^ and the study from Dische et al.^(22)^ both stated that tumor response and tissue morbidity could be compromised by deviation from the prescribed dose of 5% or more. Based on that, the difference observed in our study was not clinically significant agreeing with the conclusion of the Rana study.

The differences observed under the PTV D95% prescription were larger compared to those observed under the isocenter prescription between ${\text{AXB}}_{-}D_{m}$ and AAA, ${\text{AXB}}_{-}D_{m}$ and ${\text{AXB}}_{-}D_{w}$ and ${\text{AXB}}_{-}D_{w}$ and AAA. This is probably due to the more important difference between algorithms when it comes to the modeling of dose near the interfaces. Indeed, AAA approximates the effect of electron disequilibrium at and near the interfaces between media of different density by an empirical convolution along a ray line, resulting in the underestimation of the build‐up and build‐down effects near interfaces in the presence of very low density media like air, while AXB, that shares the same multiple‐source model as AAA but different dose calculation, has shown to better agree with measurements.^12^, ^23^

## V. CONCLUSIONS

Although statistically significant for most of the evaluated parameters, the mean difference between the $D_{w}$ and the $D_{m}$ reporting mode of the AXB in SBRT plans for lung cancer patients was within 5% points. Both dose reporting mode of the AXB seemed to agree well with the AAA, with the largest difference observed between the $D_{m}$ and the AAA.

## ACKNOWLEDGMENTS

This research was supported in part by MEXT KAKENHI Grant Number 25253078. The authors have no conflict of interest to disclose in connection with this paper.

## COPYRIGHT

This work is licensed under a Creative Commons Attribution 3.0 Unported License.
